# Clinicopathological features of breast cancer patients with internal mammary and/or supraclavicular lymph node recurrence without distant metastasis

**DOI:** 10.1186/s12885-020-07442-8

**Published:** 2020-09-29

**Authors:** Hitoshi Inari, Natsuki Teruya, Miki Kishi, Rie Horii, Futoshi Akiyama, Shunji Takahashi, Yoshinori Ito, Takayuki Ueno, Takuji Iwase, Shinji Ohno

**Affiliations:** 1 Breast Oncology Center, Cancer Institute Hospital, Japanese Foundation for Cancer Research, 3-8-31 Ariake, Koto-ku, Tokyo, 135-8550 Japan; 2 Department of Pathology, Cancer Institute Hospital, Japanese Foundation for Cancer Research, 3-8-31 Ariake, Koto-ku, Tokyo, 135-8550 Japan; 3 Department of Pathology, Cancer Institute, Japanese Foundation for Cancer Research, 3-8-31 Ariake, Koto-ku, Tokyo, 135-8550 Japan; 4 Department of Medical Oncology, Cancer Institute Hospital, Japanese Foundation for Cancer Research, 3-8-31 Ariake, Koto-ku, Tokyo, 135-8550 Japan

**Keywords:** Breast cancer, Internal mammary lymph node recurrence, Supraclavicular lymph node recurrence, Prognosis

## Abstract

**Background:**

Internal mammary and/or supraclavicular (IM–SC) lymph node (LN) recurrence without distant metastasis (DM) in patients with breast cancer is rare, and there have been few reports on its clinical outcomes.

**Methods:**

We enrolled 4237 patients with clinical stage I–IIIC breast cancer treated between January 2007 and December 2012. Clinicopathological features of patients with IM–SC LN recurrence and patients with DM were retrospectively reviewed.

**Results:**

With a median follow-up time 78 (range, 13–125) months after the primary operation, 14 (0.3%) had IM–SC LN recurrence without DM and 274 (6.5%) had DM at the first recurrence among 4237 patients. No statistical differences were found in the baseline characteristics of the primary tumor between the two groups. The 5-year overall survival (OS) rate after recurrence in patients with IM–SC LN recurrence was 51% compared with 27% in patients with DM (*P* = 0.040). In patients with IM–SC LN recurrence, clinically positive axillary LN at diagnosis and pathologically positive axillary LN at primary surgery were poor prognostic factors for distant disease-free survival (DDFS) (*P* = 0.004 and 0.007, respectively). Clinical and pathological axillary nodal status at primary surgery was associated with OS (*P* = 0.011 and 0.001, respectively).

**Conclusions:**

Patients with IM–SC LN recurrence without DM who had no clinical and pathological axillary LNs involved at primary surgery had a favorable prognosis. A larger validation study is required.

## Background

The definition of regional lymph node (LN) in breast cancer has been controversial in terms of anatomical extent. Supraclavicular LN metastasis in patients with breast cancer was classified as distant metastasis (DM) in the fifth edition of the American Joint Committee on Cancer staging manual for breast cancer, but it has more recently been classified as local disease since the sixth edition [1, 2].

Patients with internal mammary and/or supraclavicular (IM–SC) LN recurrence are reported to have better clinical outcome than those with DM. Previous studies have reported that 5-year overall survival (OS) rates after SC LN recurrence and distant recurrence were 33.6 and 9.1%, respectively [3]. However, patients with IM–SC LN recurrence have a worse clinical outcome than those with ipsilateral breast tumor recurrence [3–5].

Isolated regional LN recurrence, except for ipsilateral axillary LN recurrence, is uncommon, with a reported frequency of range 1–5.4% [6–13]. In particular, IM–SC LN recurrence without DM is rare; therefore, conducting a prospective randomized trial to compare different treatment strategies is difficult and few retrospective studies have shown long-term outcomes with IM-SC LN recurrence [3]. Thus, the clinical management of IM–SC LN recurrence without DM in patients with breast cancer is generally empirical, especially in terms of whether cure can be aimed at.

In the present study, we retrospectively reviewed data from patients with primary breast cancer who underwent surgery between 2007 and 2012 and experienced IM–SC LN recurrence and DM during follow-up. We analyzed the clinicopathological characteristics associated with survival after IM–SC LN recurrence in order to uncover groups of patients who have favorable survival outcome and, thus, may benefit from treatment at curative intent.

## Methods

### Patients

**Fig. 1:**
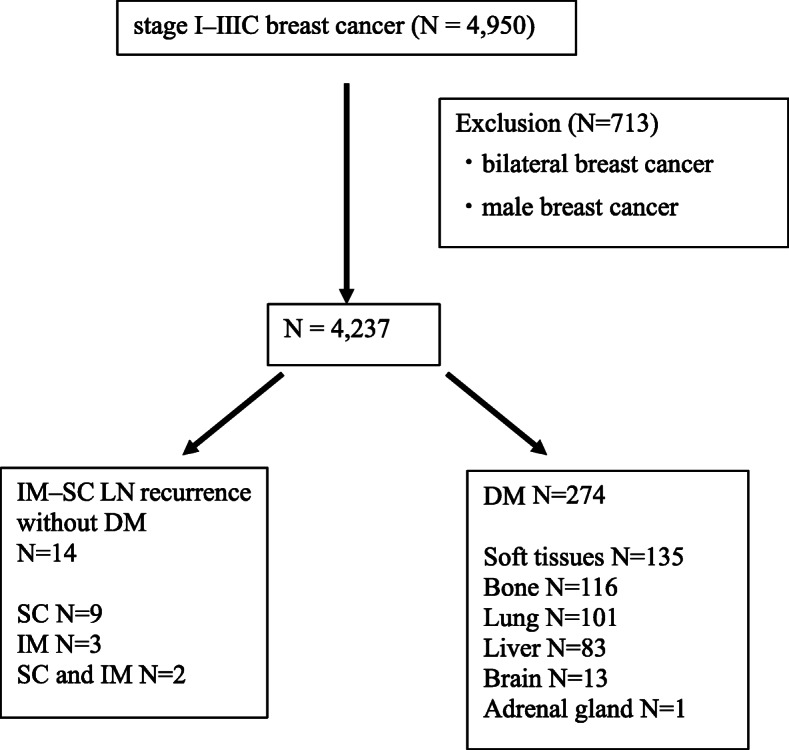
Flow diagram of the study. *DM* distant metastasis, *IM-SC* Internal mammary and/or supraclavicular, *LN* lymph node, *N* number

Data from patients treated at the Breast Oncology Center, Cancer Institute Hospital, Japanese Foundation for Cancer Research, Tokyo, between January 2007 and December 2012 were collected. Inclusion criteria included: histologically proven invasive breast cancer, clinical stage I–IIIC, those patients who received surgery from January 2007 to December 2012, and those treated at the Cancer Institute Hospital. Exclusion criteria included: bilateral breast cancer and male. Of these, 706 patients with bilateral breast cancer and seven male patients were excluded, leaving 4237 patients in this study (Fig. 1). Clinicopathological characteristics of the patients are shown in Table S1. We retrospectively reviewed the database and identified patients who experienced IM–SC LN recurrence without DM and those who experienced DM at the first recurrence during the follow-up period. The data of patients in this study are in Additional file 2.

### Definition of clinical LN status at diagnosis

All patients underwent LN evaluation by palpation and ultrasonography prior to primary surgery. Metastasis was confirmed by aspiration cytology [14].

### Adjuvant therapy

Adjuvant therapy was administered based on the guidelines provided by the Japanese Breast Cancer Society [15]. Anthracycline and/or taxane regimens were used depending on risk factors, such as tumor size, nodal status, estrogen receptor (ER) and progesterone receptor (PR) status, human epidermal growth factor receptor 2 (HER2) status, nuclear grade, and Ki-67 status. Anthracycline regimens involved 4–6 cycles of adriamycin-based or epirubicin-based regimen as described previously [16]. Taxane regimens included weekly paclitaxel or tri-weekly docetaxel [16]. Endocrine and anti-HER2 therapy was used according to the hormone receptor and/or HER2 status. Post-mastectomy radiotherapy (PMRT) was administered in patients with ≥4 positive nodes, 1–3 positive nodes with extensive lymphatic invasion, IM–SC LN metastasis, or inflammatory breast cancer. PMRT was given in the chest wall and the area of regional LNs. The prescribed dose was 50Gy in 25 fractions of 2Gy.

### Follow-up

From January 2007 to December 2015, regular postoperative palpation examinations, chest X-ray, and measurements of CEA and CA15–3 were performed every 6 months, and breast ultrasonography and mammography were performed annually. From January 2016 to October 2017 regular postoperative palpation examinations was performed every 6 months up to 5 years, and ultrasonography and mammography were performed annually up to 10 years after operation [14].

### Definition of IM–SC LN recurrence

IM–SC LN recurrence was confirmed by pathological examinations such as aspiration cytology. IM–SC LN recurrence without DM was defined as no evidence of DM at the diagnosis of IM–SC LN recurrence regardless of locoregional recurrence including ipsilateral axillary LN recurrence. A systemic survey after the diagnosis of IM–SC LN recurrence included whole-body computed tomography (CT), bone scintigraphy, and positron emission tomography/CT. The area of IM–SC LNs was determined with reference to the irradiation area of radiotherapy [17].

### Therapy of IM–SC LN recurrence without DM

Locoregional radiotherapy was indicated for patients not previously irradiated. Locoregional radiotherapy was given in the area of IM-SC LNs and/or the chest wall or breast. The prescribed dose was 50Gy in 25 fractions of 2Gy. In addition to local therapy, anthracycline and/or taxane were administered to patients who had not previously received either agent as adjuvant therapy for their primary tumor, as well as endocrine and anti-HER-2 therapy according to the tumor’s hormone receptor and HER2 status.

### Immunohistochemical analysis

Immunohistochemical analysis of ER, PR and HER2 expression was performed as described previously [18]. Samples were considered positive for ER and PR if there was a staining of ≥10% of tumor cell nuclei. Expression of HER2 was classified into four groups: 0, 1+, 2+, and 3+. Samples with 2+ expression were further tested by in situ hybridization to identify gene amplification. HER2 positivity was defined as HER2 protein 3+ or HER2 gene amplification.

### Follow-up data

Follow-up data until October 31, 2017 were collected using the database. During the study period, no patient was lost to follow-up. We retrospectively reviewed clinicopathological characteristics (including menopausal status, tumor size, LN metastasis, hormone receptor status, and HER2 status), treatment modality (surgery, chemotherapy, endocrine therapy, anti-HER2 therapy and radiotherapy), disease-free interval, IM–SC LN recurrence status (number of metastatic LNs and number of areas of metastatic LNs), and distant disease-free survival (DDFS) and OS. Pathological TNM classification was based on the Union for International Cancer Control staging system (eighth edition) [19]. DDFS was defined as the period from the day of diagnosis of locoregional recurrence until the day of diagnosis of distant metastasis or death from any cause. OS after recurrence was defined as the period from the day of diagnosis of breast cancer recurrence until the day of death from any cause. Median follow-up time was 78 (range, 13–125) months after the primary operation and 22 (range, 1–85) months after the recurrence.

We obtained informed consent from all patients, and the Ethics Committees of the institute approved the study protocol (# 2018–1100).

### Statistical analysis

Clinicopathological characteristics were compared by t-tests and chi-square tests. The Kaplan–Meier method was used to determine DDFS and OS, and survival curves were compared using the log-rank test. All *P* values were two tailed, and *P* < 0.05 was considered statistically significant. Statistical analysis was performed using IBM SPSS statistics 20 (SPSS Inc., Chicago, IL, USA).

## Results

### Clinicopathological features of patients with IM–SC LN recurrence without DM and those with DM

Among 4237 patients with breast cancer whose background characteristics are shown in Table S1, 14 (0.3%) had IM–SC LN recurrence without DM and 274 (6.5%) had DM (Fig. 1, Table 1). The median time to recurrence was 30 months in patients with IM-SC LN recurrence and 33 months in those with DM. The number of patients with different recurrence sites was shown in Fig. 1. No statistical differences were found in the baseline characteristics of the primary tumor between the two groups (Table 1). The summary of the initial treatment after IM–SC LN recurrence without DM is shown in Table S3 and S4. Surgery was performed in two patients. They underwent removal of swollen LNs in IM or SC regions for the purpose of biopsy to confirm breast cancer metastasis. No dissection of IM or SC regions was performed. One patient with IM–SC LN recurrence refused any treatment.

**Table 1:** Clinicopathological characteristics of patients with IM-SC LN recurrence and DM

Characteristics	Patients with IM-SC LN recurrence (*n* = 14)	Patients with distant metastasis (*n* = 274)	*P*-value
Disease-free survival, month (mean ± SD)	30.71 ± 6.87	33.25 ± 1.63	0.732
Age, years (mean ± SD)	45.36 ± 3.892	52.5 ± 12.93	0.093
Menopausal status at primary surgery			
Pre-	9	133	0.250
Post-	5	141	
Clinical T stage^a^			
T1	3	44	0.615
T2	9	160	
T3	2	41	
T4	0	29	
Clinical N stage^a^			
0	6	121	0.732
1	6	102	
2	1	9	
3	1	42	
Clinical stage^a^			
I	2	32	0.311
II	10	149	
III	2	93	
Perioperative chemotherapy			
No	4	52	0.376
Yes	10	222	
Surgical procedure of the primary tumor			
Partial mastectomy	4	79	0.983
Mastectomy	10	195	
Pathological LN status of the primary tumor			
Negative	7	87	0.156
Positive	7	187	
LI status of the primary tumor			
Negative	6	87	0.260
Positive	8	187	
ER status			
Positive	5	168	0.152
Negative	9	105	
HER2 status			
Positive	5	35	0.111
Negative	9	238	

^a^TNM classification is shown based on the eighth edition of the Union for International Cancer Control staging system

*DM* Distant metastasis, *ER,* Estrogen receptor, *HER2* Human epidermal growth factor receptor 2, *IM-SC* Internal mammary and/or supraclavicular, *LN* Lymph node, *LI* Lymphatic invasion, *SD* Standard deviation

**Fig. 2:**
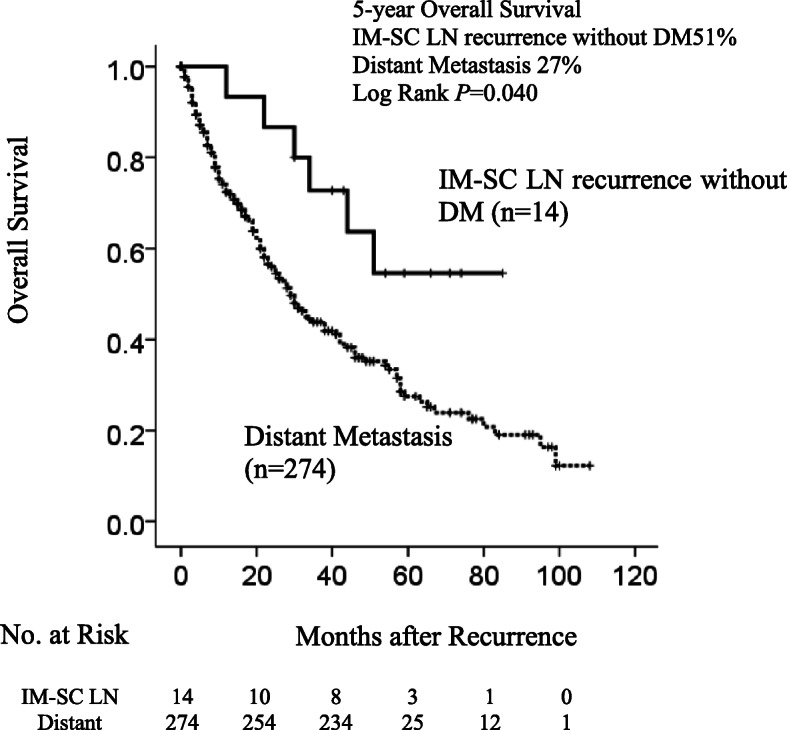
Survival Outcomes between patients with IM–SC LN recurrence without DM and those with DM. Kaplan–Meier curve for overall survival after recurrence in patients with IM–SC LN recurrence without DM (*n* = 14) and patients with DM (*n* = 274). The 5-year OS rate in patients with IM–SC LN recurrence without DM was 51% compared with 27% in patients with DM recurrence (*P* = 0.040). *DM* distant metastasis, *IM–SC LN* internal mammary and/or supraclavicular lymph node, *OS* overall survival

We examined OS after recurrence in patients with IM–SC LN recurrence without DM and those with DM (Fig. 2). The median follow-up after recurrence was 22 months. The 5-year OS rate in patients with IM–SC LN recurrence was 51% compared with 27% in patients with DM. Patients with IM–SC LN recurrence had significantly better OS than patients with DM (*P* = 0.040).

### Clinicopathological factors associated with DDFS and OS in patients with IM–SC LN recurrence

**Fig. 3:**
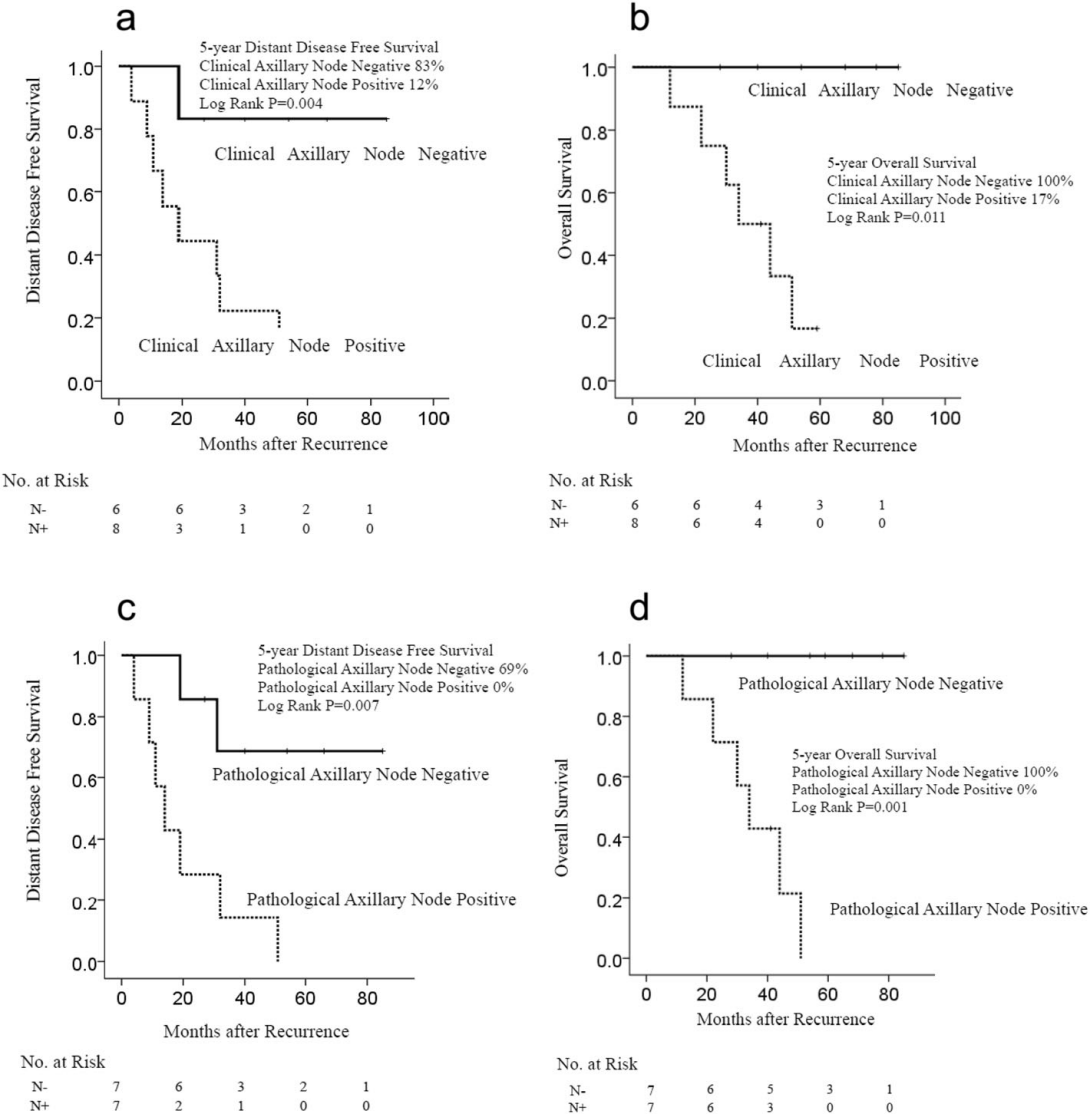
Survival Outcomes in patients with IM–SC LN recurrence without DM. Kaplan–Meier curves for DDFS (**a**) and OS (**b**) after recurrence in patients with IM–SC LN recurrence according to clinical axillary LN status of the primary tumor at diagnosis. Kaplan–Meier curves for DDFS (**c**) and OS (**d**) after recurrence in patients with IM–SC LN recurrence according to pathological axillary LN status of the primary tumor at surgery. *DDFS* distant disease-free survival, *IM–SC LN* internal mammary and/or supraclavicular lymph node, *OS* overall survival. The 5-year DDFS rates were 83% in patients with clinically axillary node-negative tumor at diagnosis and 12% in patients with clinical node-positive tumor (*P* = 0.004). The 5-year OS rates were 100% in patients with clinical axillary node-negative tumor at diagnosis and 17% in patients with clinically node-positive tumor (*P* = 0.011). The 5-year DDFS rates were 69% in patients with pathological axillary node-negative tumor at the primary surgery and 0% in patients with pathological axillary node-positive tumor (*P* = 0.007). The 5-year OS rates were 100% in patients with pathological axillary node-negative tumor at the primary surgery and 0% in patients with pathological axillary node-positive tumor at the primary surgery (*P* = 0.001)

We examined factors associated with DDFS after recurrence in patients with IM–SC LN recurrence. Prognostic factors associated with DDFS were clinical axillary LN status at diagnosis, pathological axillary LN status of the primary tumor, and PMRT (Table 2). The log-rank test showed significantly better DDFS in patients with clinical axillary node-negative tumors at diagnosis (*P* = 0.004, Fig. 3a), and OS (*P* = 0.011, Fig. 3b). Patients with pathological axillary node-negative tumor at the primary surgery showed better DDFS than those with axillary node-positive tumor (*P* = 0.007, Fig. 3c). The 5-year DDFS rate was 0% in patients with pathological axillary node-positive tumor at the primary surgery while it was 69% in those with axillary node-negative tumor. Similarly, patients with pathological axillary node-negative tumor at the primary surgery had better OS (*P* = 0.001, Fig. 3d). The 5-year OS rate was 100% in patients with pathological axillary node-negative tumor and 0% in patients with axillary node-positive tumor.

**Table 2:** Univariate analysis of prognostic factors related to DDFS in patients with internal IM-SC LN recurrence

Prognostic factor	Patients (*n* = 14)	Univariate analysis		
		HR	95% CI	*P*-value
Tumor size of primary tumor^a^				
T1 and T2	12			
T3 and T4	2	2.455	0.472–12.778	0.286
Clinical LN status at diagnosis				
Negative	6			
Positive	8	11.43	1.402–93.175	0.023
Pathological LN status of primary tumor				
Negative	7			
Positive	7	6.637	1.358–32.438	0.019
Primary ER status				
Negative	5			
Positive	9	1.031	0.271–3.929	0.964
Primary HER2 status				
Negative	9			
Positive	5	1.063	0.667–1.695	1.063
Type of surgery				
Mastectomy	10			
Partial mastectomy	4	0.570	0.116–2.792	0.570
Perioperative Chemotherapy				
Yes	10	1.724	0.356–8.355	0.499
No	4			
Post mastectomy radiation therapy				
Yes	4			
No	10	5.435	1.202–24.581	0.028
Disease free interval				
≤ 1 year	4			
> 1 year	10	0.396	0.95–1.638	0.201
Number of metastatic lymph nodes at recurrence				
≤ 2 lymph nodes	8			
≥ 3 lymph nodes	6	0.675	0.168–2.714	0.580
Number of regions of metastatic lymph nodes at recurrence				
1	12			
2	2	1.123	0.137–9.231	0.914
Hormone therapy after recurrence				
Yes	4	1.084	0.257–4.567	0.913
No	10			
Chemotherapy after recurrence				
Yes	9	0.422	0.1112–1.587	0.202
No	5			
Operation after recurrence				
Yes	2			
No	12	0.515	0.064–4.155	0.534
Radiation therapy after recurrence				
Yes	8	0.900	0.238–3.408	0.877
No	6			

^a^TNM classification is shown based on the eighth edition of the Union for International Cancer Control staging system

Under bar indicates values that are statistically significant (*P* < 0.05)

*CI* Confidence interval, *DDFS* Distant disease-free survival, *ER* Estrogen receptor, *HER2* Human epidermal growth factor receptor 2, *IM-SC* Internal mammary and/or supraclavicular, *LN* Lymph node

Because breast cancer subtypes and treatments can affect prognosis of the patients, subtypes and treatments were reviewed according to pathological LN status at primary tumor (Table S3 and S4). Surgery and chemotherapy including trastuzumab use after recurrence were different between the groups, the analysis adjusted by these confounding factors was performed as an exploratory analysis (Table S5). LN status at primary tumor remained an independent factor for DDFS after adjusting by ER, HER2, surgery and chemotherapy (*P* = 0.03) (Table S4).

## Discussion

The rate of IM–SC LN recurrence without DM was 0.3% in this study, which is concordant with other reports. The rates of isolated SC LN recurrence and IM LN recurrence were reported to be 0.4–2 and 0.08%, respectively [3, 6, 20, 21]. Because isolated IM-SC LN recurrence is very rare, it is clinically important to accumulate clinical data from different institutions to clarify an optimal treatment strategy for such rare disease.

We confirmed that patients with IM–SC LN recurrence without DM had significantly better OS after recurrence than patients with DM in agreement with the previous reports [3, 6]. We found that patients with IM–SC LN recurrence without DM had good prognosis if axillary LN was negative at the clinical diagnosis and primary surgery. Therefore, the present study suggests that some patients with IM–SC LN recurrence without DM have a favorable prognosis, particularly if axillary LNs are not involved at the clinical diagnosis and primary surgery, thus, it might be possible to consider curative treatment for patients with IM–SC LN recurrence without DM. There are several studies that examined prognostic factors after SC recurrence. Using the Danish Breast Cancer Cooperative Group treatment database, 305 patients with SC LN recurrence with or without other locoregional recurrence were identified [20]. The study showed that the combination of local and systemic treatment, negative nodal status and low grade at primary diagnosis were related to longer progression free survival after SC LN recurrence but that nodal status at primary diagnosis was not associated with OS after recurrence [20]. Another study included 42 patients with SC LN recurrence and found no association between nodal status of primary tumor and DDFS [21]. The discrepancies between studies seem to derive from the differences in perioperative systemic treatment for primary tumor and treatment strategies after regional recurrence. Indeed, these reports were based on the data from patients whose primary tumors were treated between 1977 and 2003 and between 1984 and 1994, respectively [20, 21]. In our study, the chemotherapy regimens for perioperative treatment included anthracycline and taxane and were essentially identical to the current regimens, which, we believe, makes the results more practically useful.

One of the possible explanations for poor prognosis in patients with an axillary node -positive tumor at the primary surgery is the use of adjuvant chemotherapy and PMRT at the time of primary surgery. Possibly, recurrent tumors in those with axillary node-positive disease had acquired resistance to chemotherapy and, in part, radiotherapy. On the contrary, those patients who had no axillary LN involvement of the primary tumor could receive anthracycline or taxane, or both, and radiotherapy after recurrence, which would have, to some extent, resulted in favorable prognosis. Although it is exploratory and needs cautious interpretation with this small sample size, the pathological nodal status remained prognostic for DDFS after adjusting by subtype, chemotherapy and radiotherapy (Table S5).

Our treatment strategy was consistent with the fourth ESO-ESMO International Consensus Guidelines for Advanced Breast Cancer, which recommends the use of systemic therapy (chemotherapy, endocrine therapy, and/or anti-HER2 therapy) for patients with regional recurrence, in addition to local therapy [22]. Locoregional radiotherapy was indicated for patients not previously irradiated. Previous studies showed that local and systemic combination therapy after recurrence was an independent factor for improved outcome and that patients with isolated IM LN recurrence exhibited excellent outcomes when managed with aggressive salvage treatments consisting of chemotherapy, radiation therapy and surgery [20, 23].

Curability has been uncertain; thus, the treatment strategy for IM–SC LN recurrence without DM is currently on an individual basis, either palliative or curative. Our results suggest an option for treatment strategy based on axillary nodal status at the primary surgery. Patients with IM–SC LN recurrence without axillary nodal involvement at the primary surgery may receive intensive treatment with curative intent, whereas those with axillary nodal involvement may receive palliative treatment. To confirm the clinical validity of this treatment strategy, a larger study is required.

One of the major limitations in the present study was a small number of patients, which resulted from the rarity of isolated IM–SC LN recurrence. The survival analysis of this small patient population needs to be interpreted with caution. Although we tried to validate our results using external data sources such as SEER database, the information on first recurrence sites including IM-SC LN was missing and so such analyses could not be performed [24]. More patients are necessary to confirm our results; therefore, we are planning a multicenter study focusing on prognosis of isolated regional recurrence. Another limitation was the short follow-up period. It is important to follow the patients for a longer period.

## Conclusion

We found that outcomes in patients with IM–SC LN recurrence without DM who had no axillary nodal involvement at the clinical diagnosis and primary surgery were favorable after recurrence. Therefore, the results of the present study suggest that some patients with IM–SC LN recurrence without DM can consider treatment aiming at cure if they have an axillary node-negative primary tumor.

## Supplementary information

**Additional file 1: Table S1** Clinicopathological characteristics of patients with breast cancer. **Table S3** Summary of initial treatment after IM–SC LN recurrence. **Table S4** Subtypes and treatment of patients with IM-SC LN recurrence without DM according to pathological LN status at primary tumor. **Table S5** Multivariate analysis of prognostic factors related to DDFS in patients with IM-SC LN recurrence without DM.

**Additional file 2.**

## Data Availability

The dataset supporting the conclusions of this article is included within the article (and its Additional file 2).

## Abbreviations

CT: Computed tomography
DDFS: Distant disease-free survival
DM: Distant metastasis
ER: Estrogen receptor
HER2: Human epidermal growth factor receptor 2
IM-SC: Internal mammary and/or supraclavicular
LN: Lymph node
OS: Overall survival
PMRT: Post-mastectomy radiotherapy
PR: Progesterone receptor
