# Supplementary material for: Clinicopathological features of breast cancer patients with internal mammary and/or supraclavicular lymph node recurrence without distant metastasis
Source: BMC Cancer. 2020 Sep 29;20:932. doi: 10.1186/s12885-020-07442-8 (PMC7526116; doi:10.1186/s12885-020-07442-8)
Supplement: Supplementary file 1 — Additional file 1: Table S1 Clinicopathological characteristics of patients with breast cancer. Table S3 Summary of initial treatment after IM–SC LN recurrence. Table S4 Subtypes and treatment of patients with IM-SC LN recurrence without DM according to pathological LN status at primary tumor. Table S5 Multivariate analysis of prognostic factors related to DDFS in patients with IM-SC LN recurrence without DM. [file 12885_2020_7442_MOESM1_ESM.docx]

**Additional Files**

**Table S1** Clinicopathological characteristics of patients with breast cancer

| Characteristics | Patient number = 4,237 |
| --- | --- |
| Age, years (mean ± SD) | 53.7 ± 12.0 |
| Menopausal status at primary surgery |  |
| Pre- | 2,007 |
| Post- | 2,230 |
| Clinical T stage^a^ |  |
| T1 | 2,319 |
| T2 | 1,638 |
| T3 | 166 |
| T4 | 93 |
| TX | 21 |
| Clinical N stage^a^ |  |
| 0 | 3,431 |
| 1 | 657 |
| 2 | 34 |
| 3 | 114 |
| NX | 1 |
| Clinical stage^a^ |  |
| Ⅰ | 2,177 |
| Ⅱ | 1,762 |
| Ⅲ | 298 |
| Preoperative chemotherapy |  |
| No | 3,391 |
| Yes | 846 |
| Surgical procedure of the primary tumor |  |
| Partial mastectomy | 2,507 |
| Mastectomy | 1,730 |
| Pathological LN status of the primary tumor |  |
| Negative | 2,854 |
| Positive | 1,383 |
| ER status |  |
| Positive | 3,347 |
| Negative | 744 |
| Unknown | 147 |
| HER2 status |  |
| Positive | 430 |
| Negative | 3,799 |
| Unknown | 8 |

^a^TNM classification is shown based on the eighth edition of the Union for International Cancer Control staging system

*ER* estrogen receptor, *HER2* human epidermal growth factor receptor 2, *LN* lymph node, *SD* standard deviation

**Table S3** Summary of initial treatment after IM–SC LN recurrence

| Patient | pN | CT | anti-HER therapy | ET | RT |
| --- | --- | --- | --- | --- | --- |
| 1 | negative | VNR | Tra |  | yes |
| 2 | negative | AC　PTX | Tra |  | yes |
| 3 | negative | CEF |  |  |  |
| 4 | negative | DTX | P+Tra |  |  |
| 5 | negative | PTX |  |  | yes |
| 6 | negative | AC　DTX |  |  | yes |
| 7 | negative |  |  | ANA |  |
| 8 | positive |  |  | LHRHanalog　TAM | yes |
| 9 | positive | Treatment refusal | | | |
| 10 | positive |  |  |  | yes |
| 11 | positive | VNR | Tra |  |  |
| 12 | positive | Cape |  |  | yes |
| 13 | positive |  |  |  | yes |
| 14 | positive | Cape |  |  |  |

*AC* Adriamycin cyclophosphamide, *ANA* anastrozole, *Cape* capecitabine, *CEF* cyclophosphamide epirubicin fluorouracil, *CT* chemotherapy, *DTX* docetaxel, *ET* endocrine therapy, *EXE* exemestane, *HER* human epidermal growth factor receptor 2, *IM*–*SC* internal mammary and/or supraclavicular, *LN* lymph node, *LHRH* luteinizing hormone releasing hormone, *P* pertuzumab, *pN* pathological LN status at primary tumor,*PTX* paclitaxel, *RT* radiotherapy*, Tra* trastuzumab, *TAM* tamoxifen, *VNR* vinorelbine

**Table S4** Subtypes and treatment of patients with IM-SC LN recurrence without DM according to pathological LN status at primary tumor

| Characteristics | | Pathological LN positive at primary tumor (n=7) | Pathological LN negative at primary tumor (n=7) |
| --- | --- | --- | --- |
| Subtypes | ER+ HER- | 2 | 2 |
|  | ER+HER+ | 0 | 1 |
|  | ER-HER+ | 2 | 2 |
|  | ER-HER- | 3 | 2 |
| Therapy at primary surgery | Operation |  |  |
|  | M | 5 | 5 |
|  | PM | 2 | 2 |
|  | RT |  |  |
|  | PMRT | 3 | 1 |
|  | BI | 2 | 1 |
|  | Systemic treatment |  |  |
|  | A and/or T | 6 | 4^a^ |
|  | Tra | 2 | 3 |
|  | ET | 2 | 3 |
| Initial therapy after recurrence | Operation | 0 | 2 |
|  | RT | 4 | 4 |
|  | Systemic treatment |  |  |
|  | CT | 3 | 6 |
|  | A and/or T | 0 | 5 |
|  | Tra | 1 | 3 |
|  | ET | 1 | 1 |

*A* anthracycline, *BI* breast irradiation, *CT* chemotherapy, *DM* distant metastasis, *ER* estrogen receptor, *ET e*ndocrine therapy, *HER* human epidermal growth factor receptor 2, *IM-SC* internal mammary and/or supraclavicular, *LN* lymph node, *M* mastectomy, *PM* partial mastectomy, *PMRT* post-mastectomy radiotherapy, *RT* radiation therapy*T* taxane, *Tra* trastuzumab

^a^ One patient had received both agents.

**Table S5** Multivariate analysis of prognostic factors related to DDFS in patients with IM-SC LN recurrence without DM

| Characteristics | Multivariate analysis | | |
| --- | --- | --- | --- |
|  | HR | 95%CI | *P*-value |
| Pathological LN status of primary tumor, |  |  |  |
| Negative | 1 |  |  |
| Positive | 14.63 | 1.31-163.4 | 0.03 |
| Operation after recurrence |  |  |  |
| No | 1 |  |  |
| Yes | 2.71 | 0.16-44.39 | 0.49 |
| Chemotherapy after recurrence |  |  |  |
| No | 1 |  |  |
| Yes | 0.46 | 0.36-9.17 | 1.83 |
| ER |  |  |  |
| Negative | 1 |  |  |
| Positive | 1.028 | 0.09-12.22 | 0.98 |
| HER |  |  |  |
| Negative | 1 |  |  |
| Positive | 1,68 | 0.85-3.32 | 0.13 |

Under bar indicates values that are statistically significant (*P* < 0.05).

*CI,* confidence interval, *DDFS,* distant disease-free survival, *ER* estrogen receptor, *HER* human epidermal growth factor receptor 2, *HR* hazard ratio*,IM-SC* internal mammary and/or supraclavicular, *LN* lymph node
